# Different Activation of TRAF4 and TRAF6 in Inflammatory Bowel Disease

**DOI:** 10.1155/2013/647936

**Published:** 2013-01-28

**Authors:** Jun Shen, Yuqi Qiao, Zhihua Ran, Tianrong Wang

**Affiliations:** Key Laboratory of Gastroenterology & Hepatology of Ministry of Health and Division of Gastroenterology and Hepatology, Shanghai Institute of Digestive Disease and Renji Hospital, Shanghai Jiao Tong University School of Medicine, No. 1630 Dongfang Road, Shanghai 200127, China

## Abstract

In recent years, interests combining the exploration of tumor necrosis factor receptor-associated factor 4 (TRAF4) and TRAF6 in immune cells and transgenic mice are emerging. Although it has been found that TRAF4 and TRAF6 share the same TRAF binding sites, comprehensive study of TRAF4 and TRAF6 in inflammatory bowel disease (IBD) is still lacking. This paper shows similar and different expression patterns of TRAF4 and TRAF6 in patients with IBD. The results indicate that TRAF4 and TRAF6 are overexpressed in IBD. TRAF4 and TRAF6 play different roles in the pathogenesis of IBD. Moreover, TRAF4 may be an indicator of endoscopic disease activity of UC and TRAF6 preactivation can be detected in noninflamed colonic segments.

## 1. Introduction

Tumor necrosis factor receptor-associated factors (TRAFs) act as adapter molecules controlling signaling pathways, such as nuclear factor kappa B (NF-*κ*B), interleukin-1 receptor (IL-1R), toll-like receptor (TLR), and transforming growth factor-*β* (TGF-*β*) [1, 2]. For a long time, TRAF6 has shown conserved function in activation of autoimmunity and inflammation. It is important that TRAF6 contributes to the CD40-mediated activation of NF-*κ*B and c-Jun kinase (JNK). Association of TRAF6 with CD40 is essential for CD40-mediated IL-6 expression [3], which could explain the requirement for membrane-bound CD40 ligand to induce IL-6 production by immunocytes [4]. The survival, regulation, and activation of immunocyte and epithelial cell, signaling through cell surface receptors to activate NF-*κ*B and mitogen-activated protein kinases (MAPKs) through TRAF6, are critical regulations of immune response [5]. Unlike TRAF6, the molecular mechanism of TRAF4 in multiple signaling pathways triggered by TNFR-related proteins remains enigmatic. Moreover, the subcellular localization and functions of TRAF4 have been controversial for years. It has been indicated that TRAF4 augments NF-*κ*B activation through glucocorticoid-induced TNFR (GITR) expression on T cells, B cells, and macrophages [6]. As a unique TRAF family member mediating signal transduction by TNF, IL-1R, or TLR, it is found that TRAF4 acts as a positive effector of bone morphogenetic protein (BMP) and the TGF-*β* signaling pathway [7]. 

The intestinal epithelium and immune cells in the gut establish active sites of immune reactivity. Breakdown of homeostasis between intestinal microbiota and the mucosal immune system, together with both environmental and genetic factors, leads to inflammatory bowel disease (IBD). NF-*κ*B and TLR are considered as nodal points in the suppression and/or recruitment of immune responses in IBD [8]. Interestingly, although mechanisms of TRAFs in IBD are not yet fully studied, TRAF-related inflammatory mediators as TGF-*β* or CD40 play critical roles in a wide array of cellular functions in IBD [9, 10]. 

Recent studies in immune cells and transgenic mice regarding the role of TRAF4 and TRAF6 have revealed that they share the same binding sites, yet comprehensive study of TRAF4 and TRAF6 in IBD is still lacking [11]. Based on the hypothesis that TRAF4 and TRAF6 may be activated prior to the clinical or endoscopic activation in patients with IBD, we sought to measure TRAF4 and TRAF6 expressions to explore their potential roles in IBD patients.

## 2. Materials and Methods

### 2.1. Patients and Samples

Patients were enrolled according to clinical and endoscopic diagnosis. Patients with pregnancy, colorectal resection for UC, disease involving only small bowel, poor bowel preparation as visible area of intestinal mucosa <90%, or use of steroids, immunosuppressants, or infliximab before colonoscopic sampling were excluded. No diagnosis altered after at least 3 months of followup. Endoscopic score was evaluated using simplified endoscopic score in Crohn's disease (SES-CD) or Baron score for patients with CD or UC, respectively. Healthy controls were included without sign or symptoms of bowel disease.

Human intestinal biopsies and blood samples were collected at Division of Gastroenterology and Hepatology, Shanghai Jiao-Tong University School of Renji Hospital Medicine, in accordance, with guidelines of the Research Ethics Committee of Renji Hospital, Shanghai Jiao Tong University, School of Medicine. All patients and healthy controls agreed to provide written consents. 

Human tissue specimens were taken from both macroscopically inflamed and non-inflamed regions of the colon. Biopsies from the colon of healthy donors were also analyzed. Tissue specimens were put into liquid nitrogen within 10 minutes after biopsy for protein extraction or kept in RNAlater (Qiagen) for RNA isolation. Human peripheral blood was separated into plasma and peripheral blood mononuclear cells (PBMCs). Plasma was obtained using commercially EDTA-treated tubes (Gongdong Medical Technology Co., Ltd.) and PBMCs were isolated according to Lymphoprep (Axis-Shield PoC AS, Norway) protocol. Briefly, diluted blood was overlayed over 3 mL Lymphoprep and centrifuged at 800 ×g for 20 mins. PBMCs were removed from a distinctive band at the sample interface after centrifugation. Then, PBMCs were kept in RNAlater (Qiagen) for RNA isolation according to manufacturer's protocol.

### 2.2. Enzyme-Linked Immunosorbent Assay (ELISA)

Plasma was obtained following centrifugation of whole blood for 15 minutes at 2,000 ×g. Samples were stored at −80°C prior to analysis via Elisa. Samples were analyzed using kits against TRAF4 and TRAF6, according to the manufacturer's specifications (Lanji Biochemical and Diagnostics, Shanghai, China) and a microtiter plate reader was used to read absorbance at 450 nm. Experiments were performed in triplicate.

### 2.3. RNA Isolation, cDNA Synthesis, and Real-Time PCR

Total RNA in PBMCs and tissue samples were isolated with TRIzol Reagent (Ambion) according to the manufacturer's protocol for cells and tissue. The quantity and quality of RNA were detected using a NanoDrop 1000 (NanoDrop Technologies, Wilmington, DE, USA). Primers were designed with Primer 5.0 (ABI) software and consequently synthesized by Sango Biotech (Shanghai) Co., Ltd. The primer set for TRAF4 was 5′-AGGAGTTCGTCTTTGACACCATC-3′ (forward) and 5′-CTTTGAATGGGCAGAGCACC-3′ (reverse), with a product of 162 bps. The primer set for TRAF6 was 5′-CCTTTGGCAAATGTCATCTGTG-3′ (forward) and 5′-CTCTGCATCTTTTCATGGCAAC-3′ (reverse), with a product of 140 bps. The primer set of GAPDH was 5′-GTGAAGGTCGGAGTCAACGG-3′ (forward) and 5′-CCTGGAAGATGGTGATGGGAT-3′ (reverse), which provided a product of 226 bps.

cDNAs were produced with PrimeScriptTM RT reagent Kit (Takara Biotechnology Dalian Co., Ltd.). Briefly, reverse transcripts were incubated at 37°C for 15 minutes and 85°C for 5 seconds. SYBR Premix Ex Taq kit was purchased from TakaRa and real-time PCR reactions were done using a StepOne Plus device (Applied Biosystems) at 95°C for 10 seconds followed by 40 cycles of 95°C for 5 seconds and 60°C for 20 seconds according to instruction of the SYBR Premix Ex Taq kit. The expression levels of the target genes were normalized to GAPDH with 2-ΔΔCt method [12].

### 2.4. Western Blot Analysis

For Western blot analysis, PMBCs and tissue samples were lysed in RIPA buffer (Sigma) containing protease inhibitors (Roche) and agitated on ice for 30 minutes. Protein concentrations were determined using Pierce BCA Protein Assay Kit (Pierce, Wohlen, Switzerland). Protein electrophoresis was performed according to the protocol of Mini-PROTEA III (Bio-Rad). Briefly, proteins were separated in 10% polyacrylamide gels (Tris/glycine) and transferred onto polyvinylidene fluoride membrane (Millipore). Membranes were sequentially labeled by primary and secondary antibodies. Western blots were probed with antibodies against TRAF4 (rabbit polyclonal anti-TRAF4; 1 : 1000, Santa Cruz), TRAF6 (mouse monoclonal anti-TRAF6, 1 : 1000, Santa Cruz), and *β*-actin (mouse monoclonal anti-*β*-actin; 1 : 2500, Santa Cruz). Secondary antibody was purchased from GE Healthcare life Science. Detection was enhanced by SuperSignal West Pico Chemiluminescent Substrate (Pierce). Experiments were performed in triplicate.

### 2.5. Statistical Analysis

Statistical significance was determined using GraphPad Prism 5.0 for Windows (GraphPad Software, San Diego, CA, USA). A *P* < 0.05 was considered significant with either ANOVA analysis or Tukey's multicomparison. A two-sided Fisher's exact test or *χ* ^2^ test was performed to analyze discrete variables.

## 3. Results

### 3.1. Characteristics of Included Subjects

From February 2007 to February 2010, 40 CD patients, 42 UC patients, and 40 healthy controls were included in our present study. Patients with IBD indicated significantly lower body mass index (BMI) than healthy controls (*P* < 0.0001). The UC group contained significantly more smokers than CD patients (22 : 8, *P* = 0.0023). Three patients with ileitis, 15 patients with ileocolitis, and 22 patients with colitis were enrolled in CD group. Thirteen patients with proctosigmoiditis, 20 patients with left sided colitis, and 9 patients with pancolitis were enrolled in UC group. Characteristics of included subjects were described in Table 1.

### 3.2. TRAF4 and TRAF6 Expressions in Plasma

**Figure 1:**
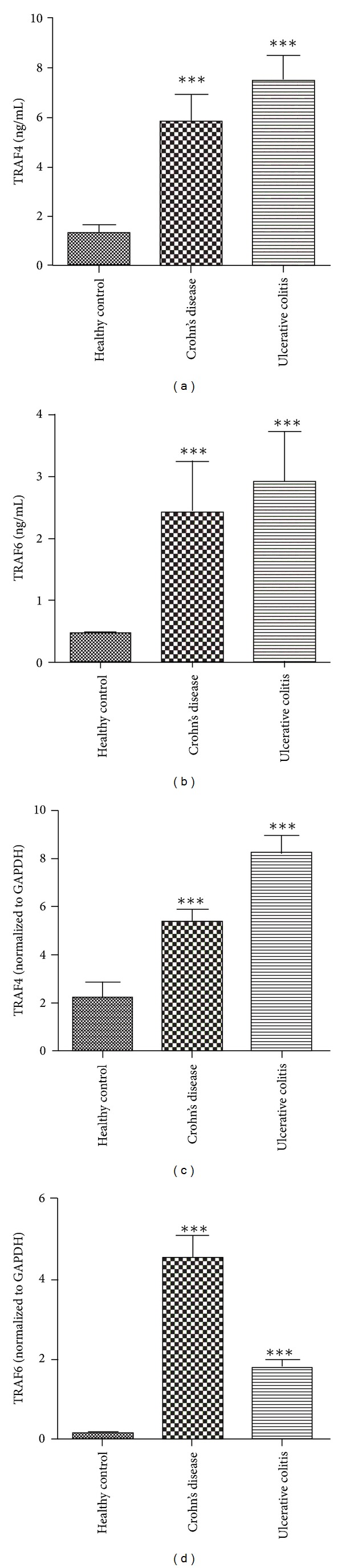
Soluble TRAF4 (a) and TRAF6 (b) protein levels in plasma; TRAF4 (c) and TRAF6 (d) gene expression in peripheral blood mononuclear cells of patients with Crohn's disease and ulcerative colitis. ***P < 0.0001.

To investigate the diagnostic value of TRAF4 and TRAF6 in IBD, we detected levels of soluble TRAF4 and TRAF6 in plasma of IBD patients. We found that TRAF4 and TRAF6 were significantly higher both in patients with CD and UC than in healthy controls (Figures 1(a) and 1(b)). However, only overexpression of soluble TRAF4 showed a significantly positive correlation with endoscopic disease activity index (Baron score) in UC patients (spearman's *r* = 0.458, *P* = 0.002).

**Figure 2:**
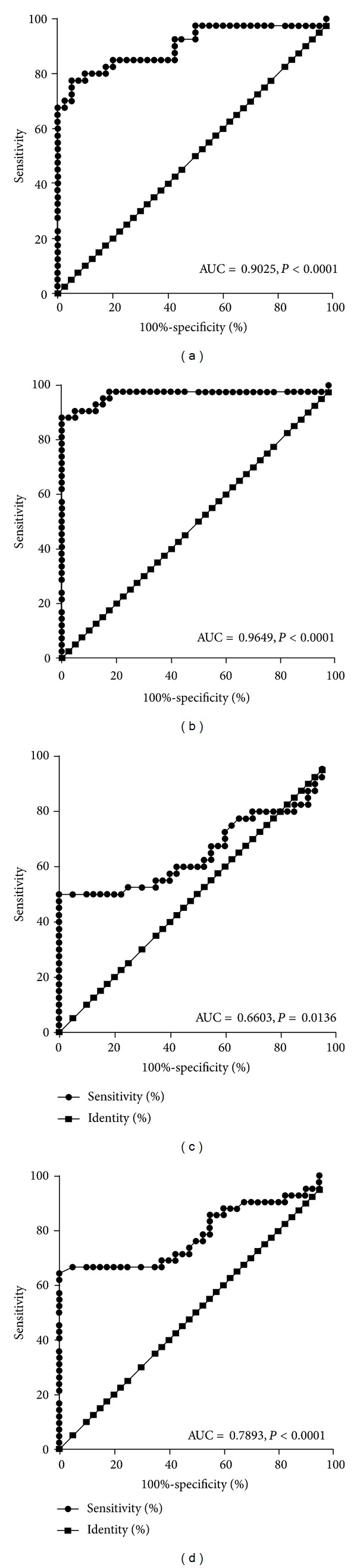
Receiver operating characteristic (ROC) curves indicate diagnostic value in differentiating active CD, UC from healthy controls. TRAF4 showed a significantly diagnostic value in differentiating active CD patients from healthy controls (a) and active UC from healthy controls (b). TRAF6 also showed a significantly diagnostic value in differentiating active CD (c) and UC (d) from healthy controls. However, the lower area under the curve (AUC) predicted a less diagnostic value than TRAF4 (c) and (d). Abbreviations: AUC: area under the curve.

Furthermore, we observed that TRAF4 showed a significantly diagnostic value in differentiating active IBD patients from healthy controls (*P* < 0.0001, Figures 2(a) and 2(b)). Although TRAF6 also showed a significantly diagnostic value in differentiating active CD, UC from healthy controls, the lower area under the curve (AUC) predicted a less diagnostic value than TRAF4 (Figures 2(c) and 2(d)). 

### 3.3. TRAF4 and TRAF6 Gene Expressions in Peripheral Blood Mononuclear Cells

To identify gene expressions of TRAF4 and TRAF6 in PBMCs in patients with CD and UC, we isolated RNA from PBMCs. Similar to their expression in plasma, TRAF4 and TRAF6 showed significantly higher levels both in patients with CD and UC than in healthy controls (Figures 1(c) and 1(d)) (all *P* < 0.0001).

### 3.4. Different Upregulation and Preactivation of TRAF4 and TRAF6 Expressions in Colonic Tissues

Given that segmental changes can exhibit inflammation in the colon in IBD patients and that intestinal segments in endoscopic remission can appear as histologic colitis, we determined the expressions of TRAF4 and TRAF6 both in inflamed and non-inflamed intestinal mucosae. Unfortunately, 3 patients with only ileitis and nine patients with pancolitis were excluded based on exclusion criteria. 5 CD patients and three UC patients were also excluded for refusal to biopsy.

**Figure 3:**
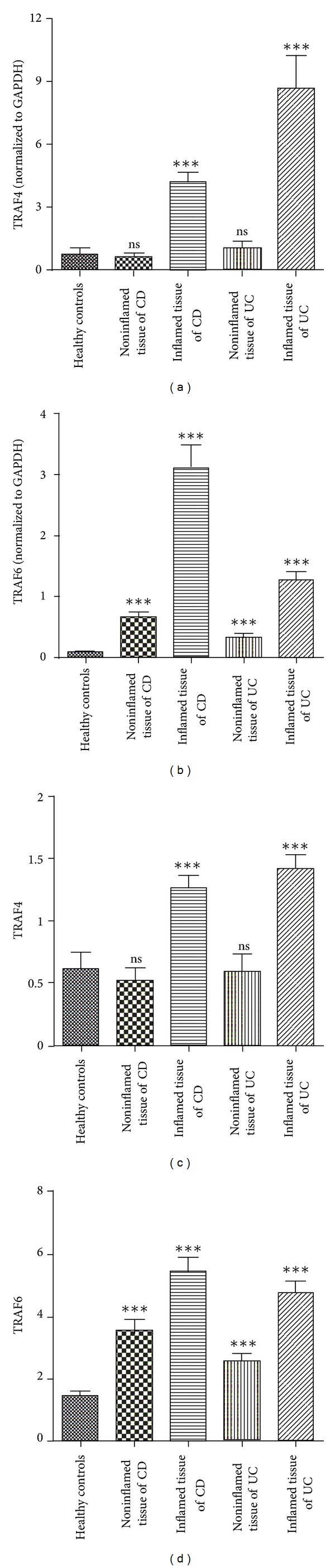
Differences in gene and protein expressions of TRAF4 and TRAF6 in the inflamed and non-inflamed colonic mucosa in patients with Crohn's disease or ulcerative colitis and in normal control tissues. (a) TRAF4 gene expression; (b) TRAF6 gene expression; (c) TRAF4 protein expression, (d) TRAF6 protein expression. ns: not significant ***P < 0.0001.

Quantitative real-time PCR (qRT-PCR) was used to determine the gene expressions of TRAF4 and TRAF6 in inflamed and non-inflamed intestinal mucosae of IBD. It was found that TRAF4 and TRAF6 expressions were significantly higher in inflamed intestinal mucosa of patients compared to normal mucosa of healthy controls (all *P* < 0.0001) (Figures 3(a) and 3(b)). Interestingly, TRAF6 expressions were also significantly higher in non-inflamed tissue of IBD patients than in healthy controls, which may indicate potential preactivation of TRAF6 in IBD (Figure 3(b)).

**Figure 4:**
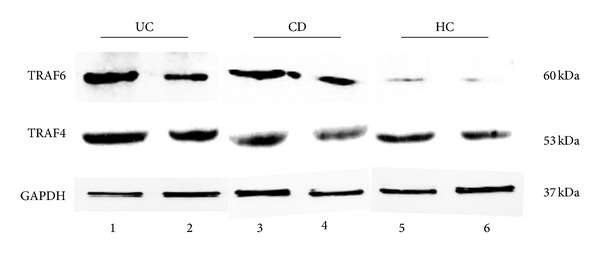
Western blot analyses of TRAF4 and TRAF6 protein expressions in colon. Abbreviations: CD: Crohn's disease; UC: ulcerative colitis; HC: healthy controls; 1, 3: inflamed colonic mucosa; 2, 4: non-inflamed intestinal mucosa under endoscopy; 5, 6: normal colonic mucosa in healthy controls under endoscopy.

Western blotting was used to measure protein expressions of TRAF4 and TRAF6 in inflamed and non-inflamed intestinal mucosae of IBD (Figure 4). Our data indicate that only TRAF6 expressions were significantly higher in non-inflamed tissue of IBD patients than in healthy controls, although TRAF4 and TRAF6 protein expressions were significantly higher in inflamed intestinal mucosa of patients than in normal mucosa of healthy controls (all *P* < 0.0001) (Figures 3(c) and 3(d)). Similar to their gene expressions, TRAF4 and TRAF6 protein expressions were significantly increased in inflamed intestinal mucosa compare to the non-inflamed mucosa or healthy controls. Also, TRAF6 protein expression was significantly higher in inflamed intestinal mucosa of IBD patients compared to healthy controls.

## 4. Discussion

In the current study, we demonstrated that two members of the TRAF family, TRAF4 and TRAF6, were activated in patients with IBD. Although both TRAF4 and TRAF6 showed potentially diagnostic value in differentiating active CD and UC from healthy controls, only TRAF6 could be pre-activated in non-inflamed tissue of IBD patients. Although TRAFs have similar overall structural features including a C-terminal receptor-binding domain and a leucine-zipper domain, their own structural difference leads to distinctive interaction with receptors [13, 14]. Growing evidence indicates that TRAFs are regulated not only by their own structural features but also by the nature of their interactions, recruitment, or localization.

The understanding of TRAF functions increased much more rapidly for TRAF6 than for TRAF4. Early overexpression studies clearly indicated that TRAF6 contributes to the CD40-mediated activation of NF-*κ*B and other signaling molecules [15]. NF-*κ*B activity has been upregulated in lamina propria immune cells and in epithelia of the inflamed gut in IBD [16]. The NF-*κ*B pathway regulates inflammation, regulatory T-cell production, and DC function. However, activation of important transcriptional regulators including NF-*κ*B and the stress-activated protein kinases (SAPKs) mediated by TRAF6 requires binding to CD40. CD40 mutants and transgenic mice have been established to address the roles of TRAF6. The cytoplasmic CD40 binding domain for TRAF6 is necessary for the CD40-mediated activation of IL-6 production in monocytes and macrophages [17], and the proinflammatory IL6 biologic network is upregulated in active IBD [18]. Moreover, experiments with immune cells or fibroblasts isolated from TRAF6-deficient mice indicate that TRAF6 is required for the CD40-mediated activation of not only NF-*κ*B but also JNK and p38 MAPK signals [19, 20]. Although CD40-mediated JNK activation in B cells seems to require cytoplasmic TRAF6, TRAF6 mutants defective in CD40 binding were able to activate the JNK pathway and upregulate CD80, indicating that TRAF6 may be able to contribute to certain JNK signals without the binding of CD40 [21]. JNK and MAPK signaling pathways are involved in governing lymphocyte influx into the gut in IBD patients by regulating lymphocyte adhesion and transmigration [22]. Furthermore, only one study screened potential alterations of the TRAF6 gene in a large number of CD and UC patients but failed to identify apparent disease-causing mutations [23]. Their data suggested that TRAF6 may have essential roles in human biology that it might not tolerate any significant structural alterations.

TRAF4 possesses several unique characteristics that are different from other members of TRAFs. The subcellular localization and molecular mechanisms of action of TRAF4 are controversial. Few studies have implicated TRAF4 in IBD or colitis. Dendritic cells from TRAF4-deficient mice exhibited reduced migration both *in vitro* and *in vivo* experiments [24]. This result indicates that TRAF4 could participate in immune functions by facilitating immune cell migration. Interestingly, TRAF4 increases NF-*κ*B activation through the GITR via a TRAF-binding site located in the cytoplasmic domain of GITR. This domain is responsible for the inhibition of Treg cells and the promotion of T-cell activation. Although no NF-*κ*B activity has ever been detected via TRAF4 overexpression, TRAF4 has been implicated as an upstream molecule that regulates the JNK pathway via interaction and activation of Misshapen (Msn), a member of the SPS1 protein kinase family [25]. Moreover, TRAF4 positively regulates transforming growth factor (TGF)-*β* via potentiating bone morphogenetic protein (BMP) and Nodal signaling [26]. TGF-*β* plays a role in the pathogenesis of IBD by activating its specific receptors [27]. All TRAFs except TRAF4 have been identified to directly or indirectly act with CD40. Accordingly, TRAF4 is upregulated in B cells following CD40 signaling, which suggests that TRAF4 affected downstream of CD40 pathway [28]. This may explain our result that TRAF4 expressions were not significantly different in non-inflamed tissue of IBD patients compared to healthy controls. 

Our understanding of TRAF4 and TRAF6 functions in the present study indicates that overlapping and unique functions will ultimately be attributed to each of the TRAFs. To our knowledge, this paper may be the first one to combine analyzing TRAF4 and TRAF6 in IBD patients. However, there are still some limits to the present study. First, the present study did not include histological criteria to distinguish non-inflamed tissue from inflamed tissue, which may confuse the microscopically inflamed tissues with non-inflamed tissues. Second, the present study could not include laboratory data as ESR or CRP to assess activity of disease, which may lead to the potential bias of overreliance on clinical manifestations and endoscopic assessment. Collectively, our data showed similar and different expression patterns of TRAF4 and TRAF6 in patients with IBD. To date, correlations between TRAF4 and TRAF6 have not been explored to elucidate signaling cascades. Future studies to determine multifaceted roles may offer targets in the treatment of IBD.

## Figures and Tables

**Table 1:** Characteristics of included subjects.

	CD (*n* = 40)	UC (*n* = 42)	HC (40)
Gender (female/male)	21/19	20/22	20/20
Age (years)	33.58 (28.87–38.28)	41.64 (37.18–46.10)	35.48 (30.06–40.89)
BMI (kg/m^2^)	19.29 (18.81–19.76)***	20.00 (19.41–20.59)***	22.84 (22.10–23.58)
Smoking (yes/no)	8/32	22/20**	3/37
Extent			
Ileitis	3		
Ileocolitis	15		
Colitis	22		
Proctosigmoiditis		13	
Left-sided colitis		20	
Pancolitis		9	
Therapy			
5-ASA/SASP	36	42	
Glucosteroids	22	16	
AZA	8	6	
Infliximab	6	1	
Surgery	1	1	
Endoscopic score^†^	3.200 (1.650–6.000)	2.000 (1.000–3.000)	

CD: Crohn's disease; UC: ulcerative colitis; HC: healthy controls; BMI: body mass index; 5-ASA: 5-aminosalicylic acid; SASP: sulfasalazine; AZA: azathioprine; ***P < 0.0001, significance is the difference from healthy controls; **P < 0.01, significance is the difference from patients with Crohn's disease; ^†^values are medians and 25%–75% percentile, simplified endoscopic score in Crohn's disease (SES-CD) is used to validate endoscopic severity in Crohn's disease, and Baron score is used to validate endoscopic severity in ulcerative colitis, respectively.
